# Residue-Level Contact Reveals Modular Domain Interactions of PICK1 Are Driven by Both Electrostatic and Hydrophobic Forces

**DOI:** 10.3389/fmolb.2020.616135

**Published:** 2021-01-27

**Authors:** Amy O. Stevens, Yi He

**Affiliations:** Department of Chemistry and Chemical Biology, The University of New Mexico, Albuquerque, NM, United States

**Keywords:** PICK1, inter-domain dynamics, coarse-grained simulations, key residues, physical forces

## Abstract

PICK1 is a multi-domain scaffolding protein that is uniquely comprised of both a PDZ domain and a BAR domain. While previous experiments have shown that the PDZ domain and the linker positively regulate the BAR domain and the C-terminus negatively regulates the BAR domain, the details of internal regulation mechanisms are unknown. Molecular dynamics (MD) simulations have been proven to be a useful tool in revealing the intramolecular interactions at atomic-level resolution. PICK1 performs its biological functions in a dimeric form which is extremely computationally demanding to simulate with an all-atom force field. Here, we use coarse-grained MD simulations to expose the key residues and driving forces in the internal regulations of PICK1. While the PDZ and BAR domains do not form a stable complex, our simulations show the PDZ domain preferentially interacting with the concave surface of the BAR domain over other BAR domain regions. Furthermore, our simulations show that the short helix in the linker region can form interactions with the PDZ domain. Our results reveal that the surface of the βB-βC loop, βC strand, and αA-βD loop of the PDZ domain can form a group of hydrophobic interactions surrounding the linker helix. These interactions are driven by hydrophobic forces. In contrast, our simulations reveal a very dynamic C-terminus that most often resides on the convex surface of the BAR domain rather than the previously suspected concave surface. These interactions are driven by a combination of electrostatic and hydrophobic interactions.

## Introduction

**Figure 1:**
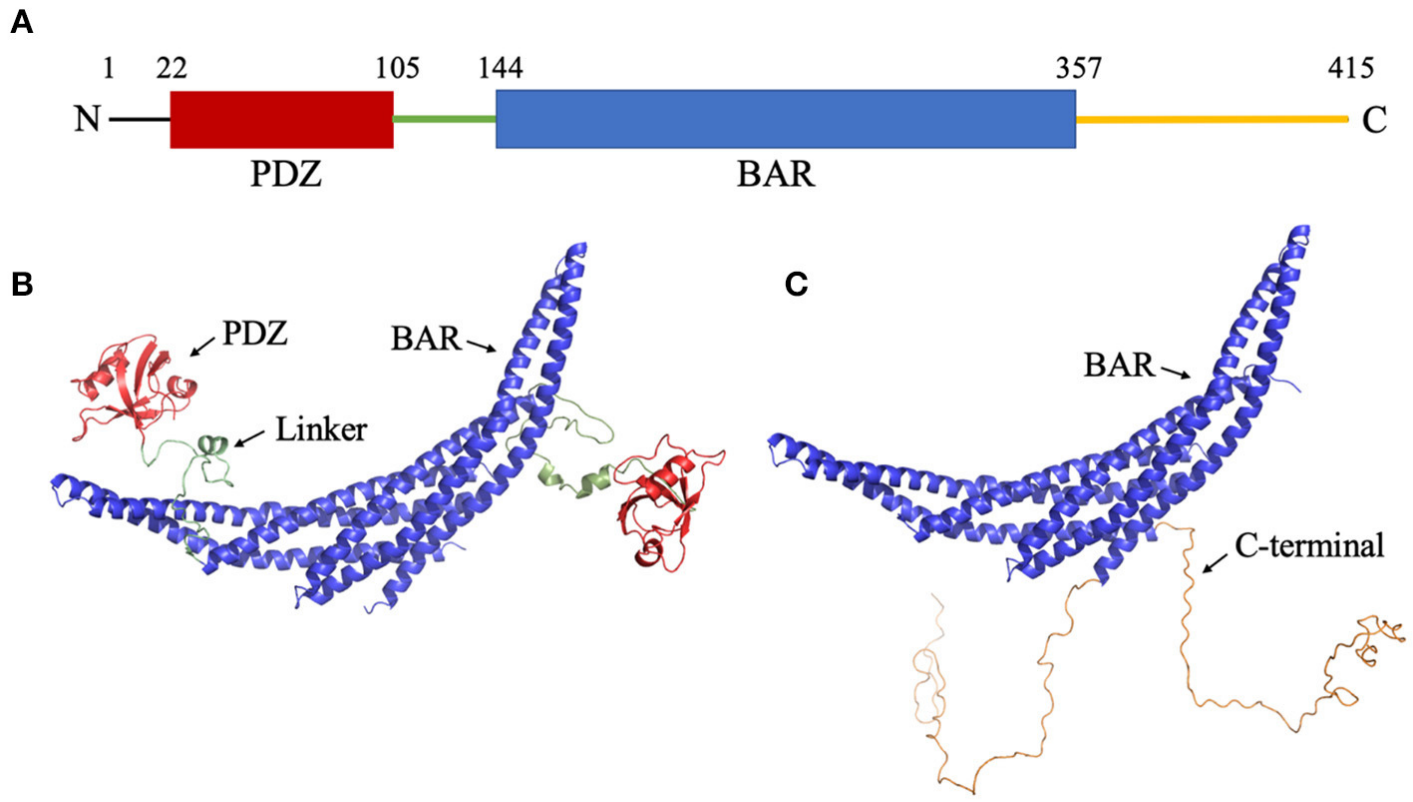
Structure of PICK1. **(A)** Sequence of PICK1. Monomeric PICK1 is comprised of two modular domains, PDZ (red) and BAR (blue), and three intrinsically disordered regions, N-terminal, linker (green), and C-terminal (yellow). **(B)** Structure of PICK1 in the absence of N- and C-termini. **(C)** Dimeric BAR domain and intrinsically disordered C-terminal.

Protein Interacting with C Kinase-1 (PICK1) is a multi-domain mammalian membrane protein (Staudinger et al., 1995). In the monomeric form, PICK1 is comprised of one PDZ (PSD-95/Dlg1/ZO-1) domain (Sheng and Sala, 2001; Hung and Sheng, 2002) and one BAR (Bin/amphiphysin/Rvs) domain (Takei et al., 1999). While each is a common modular domain, PICK1 is unique as it is the only known protein that contains both a PDZ and a BAR domain. The domains are connected via an intrinsically disordered linker that allows the PDZ domain to have a wide range of motion around the BAR domain. This range of motion increases the effective concentration of PDZ domain so that it can form protein-protein interactions with a variety of cellular proteins. Furthermore, the N- and C-termini are intrinsically disordered regions that may be involved in the regulation mechanism of PICK1. The short N-terminus (~18 residues) sits before the PDZ domain and is enriched with many acidic residues. The lengthy C-terminus (~60 residues) follows the central BAR domain and is characterized by a stretch of acidic residues. The structure of PICK1 is shown in Figure 1. Functionally, PICK1 is involved in the trafficking of a variety of proteins, including receptors, transporters, and ionic channels (Staudinger et al., 1997; Torres et al., 1998, 2001; Dev et al., 1999; Boudin et al., 2000; Cowan et al., 2000; El Far et al., 2000; Takeya et al., 2000; Jaulin-Bastard et al., 2001; Lin et al., 2001a,b; Penzes et al., 2001; Duggan et al., 2002; Hruska-Hageman et al., 2002; Perroy et al., 2002; Enz and Croci, 2003; Hirbec et al., 2003; Leonard et al., 2003; Williams et al., 2003; Meyer et al., 2004; Reymond et al., 2005). Its wide range of functions in regulating membrane proteins has drawn attention as a possible drug target. PICK1 has been identified as a possible target in ischemia (Dixon et al., 2009), Alzheimer's disease (Alfonso et al., 2014), Parkinson's disease (He et al., 2018), chronic pain (Garry et al., 2003), and cocaine addiction (Jensen et al., 2018). If PICK1 is to be targeted with the necessary affinity and specificity, an in-depth understanding of the activation mechanism and protein-protein interactions of PICK1 are vital.

PICK1 interacts with the final C-terminal residues of receptors, transporters and transmembrane channels via its PDZ domain (Hanley, 2008). The PICK1 PDZ domain has a well-defined binding pocket with canonical Class II ligand-PDZ interactions (Madsen et al., 2005). PICK1 regulates the trafficking of membrane proteins via electrostatic interactions between the membrane and the dimeric BAR domain. The family of BAR domain proteins is one of the largest groups of membrane curving proteins in the cell. The amphiphysin BAR domain binds to the negatively charged lipid membrane via two pairs of positively charged residues (Peter et al., 2004). Sequence alignment with the amphiphysin BAR domain suggests that five positively charged residues (K251, K252, K257, K266, and K268) on the PICK1 BAR domain are responsible for its interactions with the lipid membrane (Xu and Xia, 2007). Point mutation analysis further confirms the importance of these residues in lipid membrane binding (Jin et al., 2006). While an atomic-level understanding of these processes remains unclear, detailed hypotheses of auto-inhibition exist. Jin et al. used truncated mutants of PICK1 to test their lipid-binding capabilities (Jin et al., 2006). It was shown that the deletion of the C-terminus promotes BAR interactions with the lipid membrane. Results affirmed that PICK1 is negatively regulated by its C-terminus and positively regulated by its linker and PDZ domain (Jin et al., 2006). Furthermore, it is suggested that the negatively charged region of the C-terminus negatively regulates the function of PICK1 by interacting with and thus covering the critical positively charged residues on the concave surface of the BAR domain.

Our previous work has shown that the PDZ domain forms interactions with the BAR domain, which may prevent the binding between the BAR domain to the lipid membrane (He et al., 2011). These results support the hypothesis of an inactivated state of PICK1 in which ligand binding results in activation via a conformational change to expose the BAR domain to the membrane (Lu and Ziff, 2005; Rocca et al., 2008). A more recent experiment has revealed a more dynamic pattern for the interactions between the BAR and PDZ domains (Karlsen et al., 2015). Small-angle X-ray scattering (SAXS) analysis revealed the wide range of flexibility of the PDZ domain via the intrinsically disordered linker. Higher-order oligomeric structures of PICK1 further enable the dynamic positioning of the PDZ domains (Karlsen et al., 2015). Moreover, several experiments done by different groups show that the linker of the PICK1 protein may play a key role in promoting BAR interactions with the lipid membrane (Jin et al., 2006; Herlo et al., 2018). To understand the interplay between different parts of the PICK1 protein in its biological dimeric form, dynamics information at residue resolution and a very fine time resolution (picosecond or nanosecond time scale) is essential. However, such dynamics information is difficult to obtain from experiments since PICK1 is inside of the cell and forms aggregates with itself.

PICK1 is a large protein and performs its biological function in dimeric form. Such a system consists of more than 800 residues and may have a dimension over 20 nm because of its flexibility (Karlsen et al., 2015). A system of this size is extremely computationally demanding to simulate with all-atom force fields. Physics-based coarse-grained models have a long history of helping scientists to tackle systems of this size with reasonable computational resources. The physics-based UNited-RESidue (UNRES) (Liwo et al., 1997a) force field, which was originally proposed by Liwo and Scheraga, is one of the extensively tested coarse-grained models that can be used to predict protein structure (He et al., 2009, 2019) and probe large protein dynamics (He et al., 2011; Gołaś et al., 2012; Mozolewska et al., 2015). With several generations of optimization, UNRES is a reliable tool to explore the inter-domain dynamics of PICK1.

Here, we present the needed structural and dynamics information that is responsible for the auto-inhibition of PICK1 and provides a complete picture of the inter-domain dynamics of PICK1. We implemented coarse-grained UNRES molecular dynamics simulations to model two systems: (1) BAR domain with PDZ domain and linker and (2) BAR domain with C-termini. These truncations are modeled after experimental work (Jin et al., 2006) that describes systems (1) and (2) as the two extreme cases of the enhanced and reduced biological function of PICK1, respectively. The truncated systems allow us to more readily isolate the key interactions in each of these extreme cases. Our results show that the PDZ domain and linker form dynamic interactions on the concave surface and side of the BAR domain dimer. The PDZ domain interacts with the BAR domain dimer via residues that are located in the regions which are regulated by the electrostatic allosteric effects upon the formation of the PDZ-ligand complex. Surprisingly, our results do not show the C-termini interacting with the concave surface of the BAR domain via electrostatic interactions as previously expected. Rather, the movements of the C-termini are vastly dynamic and generally reside at the central region of the convex surface of the BAR domain.

## Methods

Though the experimental structures of PICK1 and the PICK1 dimer have not yet been determined, the PICK1 BAR domain has a high sequence identity with Arfaptin-2, a N-BAR domain protein (Nakamura et al., 2012). The dimer structures of N-BAR domains have been well-established. The starting structures used in the simulations were created using the BAR dimer in Arfaptin-2 as a template to create the PICK1 dimeric BAR domains using MODELER (Šali and Blundell, 1993; Fiser et al., 2000; Martí-Renom et al., 2000; Webb and Sali, 2016). With the BAR dimer, the structure of the C-termini of PICK1 was randomly generated and attached to the BAR domain. The structure of the PDZ domain has been previously experimentally determined (Pan et al., 2007) and was used as a structural template in our protocol. After the PDZ domains were randomly placed with respect to the BAR domain, the intrinsically disordered linker was added to connect the PDZ domain and BAR domain. The initial structures are shown in Figures 1B,C.

UNRES (Liwo et al., 1997a,b, 1998, 2011; He et al., 2009; Sieradzan et al., 2014) uses a simplified representation in which a protein chain is composed of a sequence of α-carbon atoms connected by virtual bonds with attached side chains. To reduce computational cost and maintain residue-level resolution, each residue is represented by two interaction sites. One interaction site is centered between two consecutive Cα atoms, and the other is located at the center of the mass of the corresponding side chain. As a physics-based coarse-grained force field, the UNRES energy function has been averaged over the lost degree of freedom when simplifying from all-atom to coarse-grained representations. Recently, UNRES has been expanded to include both nucleic acids and lipid membranes (He et al., 2013; Sieradzan et al., 2018; Ziȩba et al., 2019). Canonical MD simulations (13 trajectories) were carried out for each complex to explore the interplay between the different parts of PICK1 and the crescent BAR domains. The most recently parameterized UNRES force field (Sieradzan et al., 2017; Lubecka et al., 2019), which has been evaluated based on CASP 13 targets, was used in this work.

The input files (including all input parameters) were generated using the UNRES server at http://unres-server.chem.ug.edu.pl. While the UNRES server was used to generate input files for each system, simulations were performed locally because the UNRES server has a size limit that is smaller than the system sizes explored in this work. The simulations used the latest UNRES source code that can be downloaded at https://unres.pl/downloads. The input files generated by the UNRES server used the most recent UNRES force field, namely “NEWCT-9P = JCP 150 155104 (2019).” Users must click the “advanced” button (after selecting “MD” option) located at the top-right of the web page to use this force field. Both systems started from the PDB structures described above with periodic boundary conditions set at 10,000.0 Angtroms. No secondary structure restraints were applied. Distance restraints were manually added to the input files generated by the UNRES server to maintain the structure of the BAR and PDZ domains but not the linker or the C-terminus. It should be noted that the UNRES server does not include keywords to add distance restraints. Since PICK1 is much larger than the proteins used to parametrize the force field, a higher temperature (350K) was used for all canonical MD simulations of the two systems simulated. It should be noted that the 350K used here does not directly correspond to 350K in a biological system. Rather, a temperature of 350K is used to estimate a temperature between 300K and 350K in a biological system based on the evaluation of the previous work (Liwo et al., 2019). The time increment for integrating the equations of motion δt was 9.78 fs. Thirteen trajectories were carried out, and each trajectory has 80,000,000 steps. Snapshots of structures are outputted every 10,000 steps. Our input files of all systems have been included in the supporting information. All other parameters are default values provided by the UNRES server.

## Results

**Figure 2:**
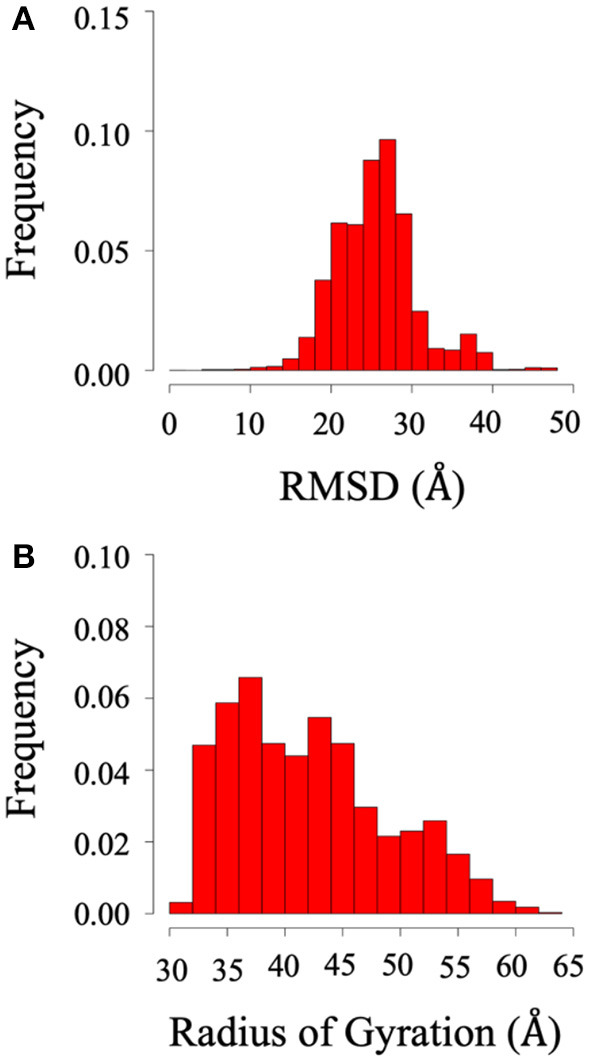
RMSD and radius of gyration of System 1 (BAR domain with PDZ domain and linker). **(A)** RMSD. **(B)** Radius of gyration. The wide range of frequency signifies the system is very dynamic.

Root mean square deviation (RMSD) and radius of gyration (Rg) analysis were performed to quantify the flexibility of the dynamic system. Frequency refers to the proportion of frames with the given distance. Figure 2 shows the RMSD and radius of gyration calculated using all the trajectories of System 1 (the BAR domain with the PDZ domain and linker). The RMSD plot (Figure 2A) shows a median RMSD at ~28 Å. This is a significant variation from the initial structure. Furthermore, the radius of gyration analysis supports these results as the size of the protein fluctuates between 30 and 60 Å with relatively significant frequencies. This analysis reveals the wide range of motion of the PDZ domain about the BAR domain as a result of the flexible linker.

**Figure 3:**
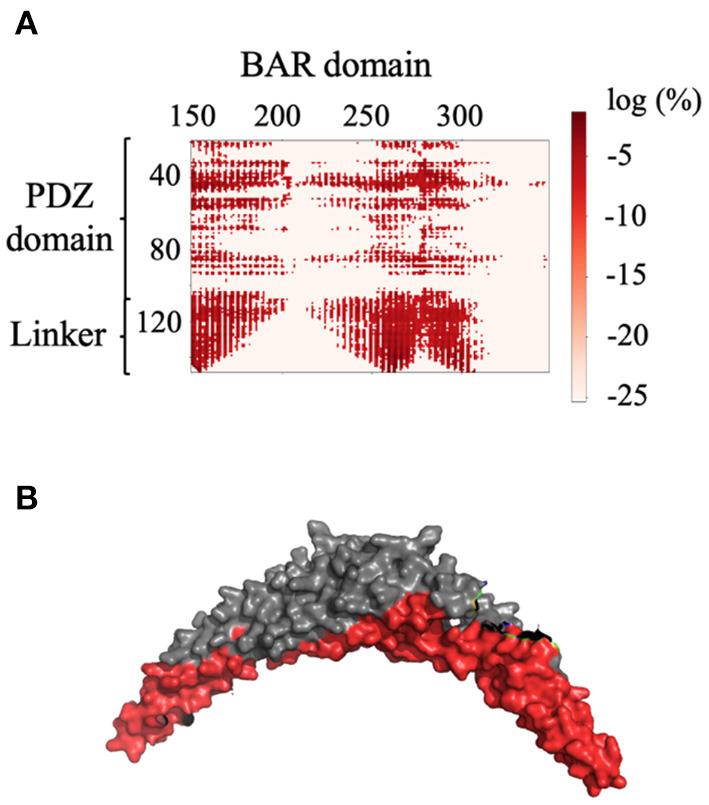
Contact between the BAR domain dimer and the PDZ domain and linker. **(A)** Contact map of BAR domain with PDZ domain and linker region. PDZ domain and linker region interact with approximate residues 150–200 and 250–300 of the BAR domain. **(B)** Dimeric BAR domain with residues 150–200 and 250–300 colored red. The color bar describes the probability of the contact as the log of the percentage of frames that the contact occurs.

For each of the two systems, contact maps were used to reveal the major interactions between any pair of residues. Contact was defined as any two Cα atoms at least five residues apart with a distance separation of 8Å or less. In System 1, the PICK1 complex is in the proposed inactivated state as the protein was neither in complex with ligand nor in proximity with the lipid membrane. As expected for inactivated PICK1, the PDZ domains formed contact with a wide range of residues located on the concave face and side surface of the BAR domains, as seen in Figure 3. Figure 3A describes the contact between the BAR domain and the PDZ domain and the BAR domain and the intrinsically disordered linker. Both the PDZ domain and the linker form the majority of interactions with residues 150–200 and 250–300 of the BAR domain. Figure 3B highlights these regions of residues on the BAR domain dimer. The PDZ domain and linker region reside near the concave surface of the BAR domain dimer in the inactivated state of PICK1. Though there are extensive interactions between PDZ and BAR domains, none of the interactions appear in >10% of the frames in the combined trajectories. This agrees with previous experimental observations that suggest a dynamic interaction pattern between the PDZ and BAR domains.

Detailed residue-residue interaction analysis revealed that the short helical portion of the linker region forms significant interactions with the PDZ domain. Key residues of PDZ-linker interaction were elucidated by identifying the most prevalent contacts, in this case, forming a contact in 13% of the frames. Ten key interaction pairs were identified between the PDZ domain and the linker, as shown in Table 1. It is not surprising that the linker can form significant contact with the PDZ domain of PICK1 as the linker and PDZ domain are next to each other in sequence. It should be noted that all listed contacts in Table 1 are formed between the helical fragment of the linker and the PDZ domain. Previous work has highlighted the importance of the helical fragment in the linker region in assisting the alignment of the BAR domain to the membrane (Herlo et al., 2018). The linker may compete with the BAR domain to interact with the PDZ domain in the inactivated PICK1 dimer.

**Table 1:** Interacting residue pairs between PDZ and Linker.

**Residue 1**	**Residue type**	**Residue 2**	**Residue type**	**Probability (%)**	Lifetime^*^
50	VAL	114	LEU	21.1	1.70 ± 1.31
66	ALA	114	LEU	17.2	1.80 ± 1.65
43	TYR	113	SER	16.6	1.59 ± 1.14
41	ALA	117	VAL	13.7	1.67 ± 1.19
43	TYR	117	VAL	13.6	1.69 ± 1.16
66	ALA	112	MET	13.5	1.68 ± 1.38
43	TYR	112	MET	13.1	1.55 ± 1.27
50	VAL	118	LEU	13.1	1.67 ± 1.34
66	ALA	113	SER	13.1	1.64 ± 1.19
50	VAL	113	SER	13.0	1.58 ± 1.18

* *unit is 100,000 UNRES simulation steps*.

**Figure 4:**
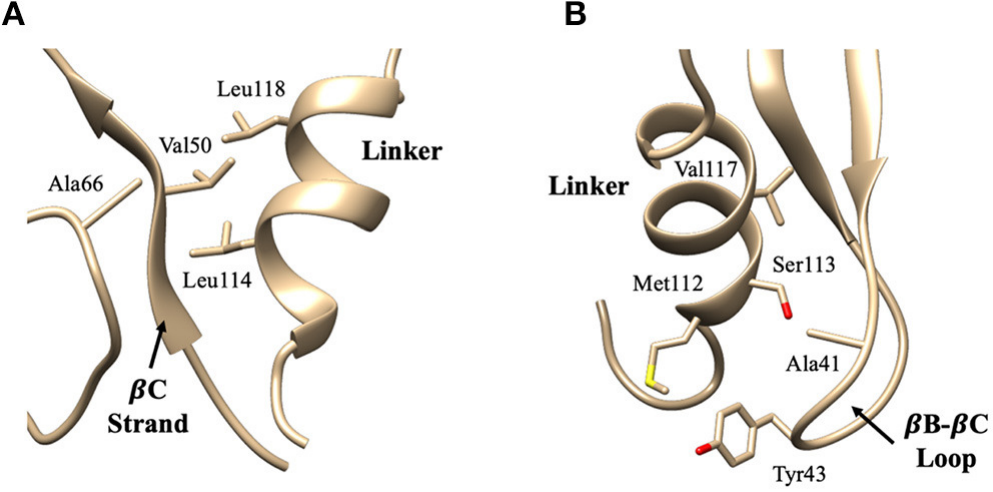
Key interaction pairs between the PDZ domain and the linker. **(A)** shows the hydrophobic core forming between the PDZ βC-strand/αA-βD loop and the short helix of the linker. Key interaction pairs Val50-Leu114 and Ala66-Leu114 listed in Table 1 can be visualized in **(A)**. **(B)** shows the hydrophobic core forming between the PDZ βB-βC loop and the short helix of the linker. Key interaction pairs Tyr43-Ser113 and Ala41-Val117 listed in β can be visualized in **(B)**.

These ten key pairs are hydrophobic interactions between the βB-βC loop, βC strand, and αA-βD loop of the PDZ domain and the short helix fragment of the linker region, as shown in Figure 4. It can be seen that the PDZ domain forms a group of hydrophobic interactions surrounding the hydrophobic helical fragment in the linker region. It has been shown that this short helical region in the linker is critical for the biological function of the BAR domain (Herlo et al., 2018). Our results suggest that this linker may mediate and/or regulate the interactions between the PDZ and the BAR domains. In addition to the frequency of each contact pair, the lifetime of each pair has also been investigated. The lifetime was calculated based on the lasting time of each contact. Since it is difficult to directly connect UNRES simulation steps to the real world time scale, lifetime is defined directly using UNRES steps. While all contacts shown in Table 1 have a probability larger than 13%, their lifetime is rather short compared to PDZ and BAR domain interactions. This may be due to the flexible nature of the linker region.

**Figure 5:**
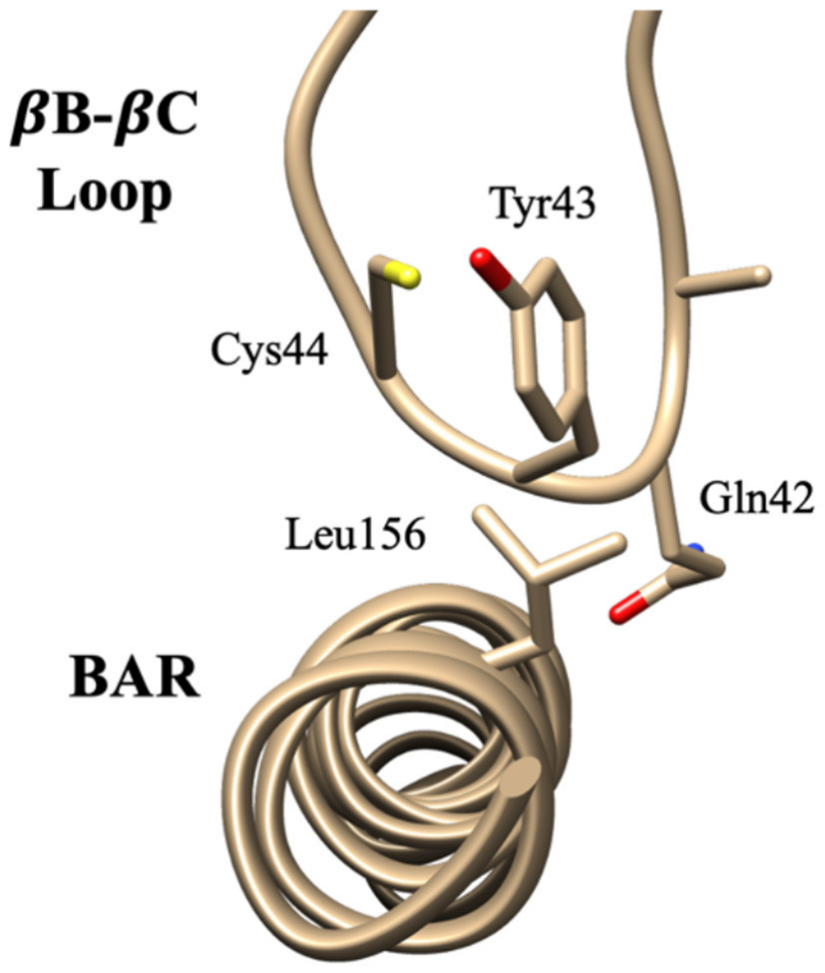
Key interaction pairs between the PDZ domain and the BAR domain. Visualization of key interaction pairs between the PDZ βB-βC loop and the BAR domain (Gln42-Leu156, Tyr43-Leu156, and Cys44-Leu15) listed in Table 2.

The interaction pattern between the PDZ and the BAR domains is quite different than the interactions between the PDZ domain and the linker. The top ten contact residue pairs are shown in Table 2. The three most probable interaction pairs (probability >7%) are between the βB-βC loop of the PDZ domain and the BAR domain, as shown in Figure 5. Though the probability of each of the ten pairs is below 10%, the lifetime of these interactions is much longer than the lifetime of the PDZ-linker interactions. These results suggest that the PDZ and BAR domain interactions are more stable than the PDZ-linker interactions despite lower probabilities.

**Table 2:** Interacting residue pairs between PDZ and BAR.

**Residue 1**	**Residue type**	**Residue 2**	**Residue type**	**Probability (%)**	Lifetime^*^
44	CYS	156	LEU	8.5808	4.28 ± 5.65
42	GLN	156	LEU	7.490471	3.95 ± 5.44
43	TYR	156	LEU	7.446148	6.46 ± 11.89
130	SER	608	SER	6.905416	3.01 ± 3.25
44	CYS	152	ARG	6.205124	3.68 ± 4.63
43	TYR	153	LEU	5.983512	3.90 ± 4.73
42	GLN	160	ALA	5.983512	2.06 ± 1.88
54	ASP	156	LEU	5.921461	4.61 ± 9.70
42	GLN	258	PHE	5.673256	3.79 ± 4.38
84	VAL	265	LEU	5.575747	3.84 ± 3.89

* *unit is 100,000 UNRES simulation steps*.

**Figure 6:**
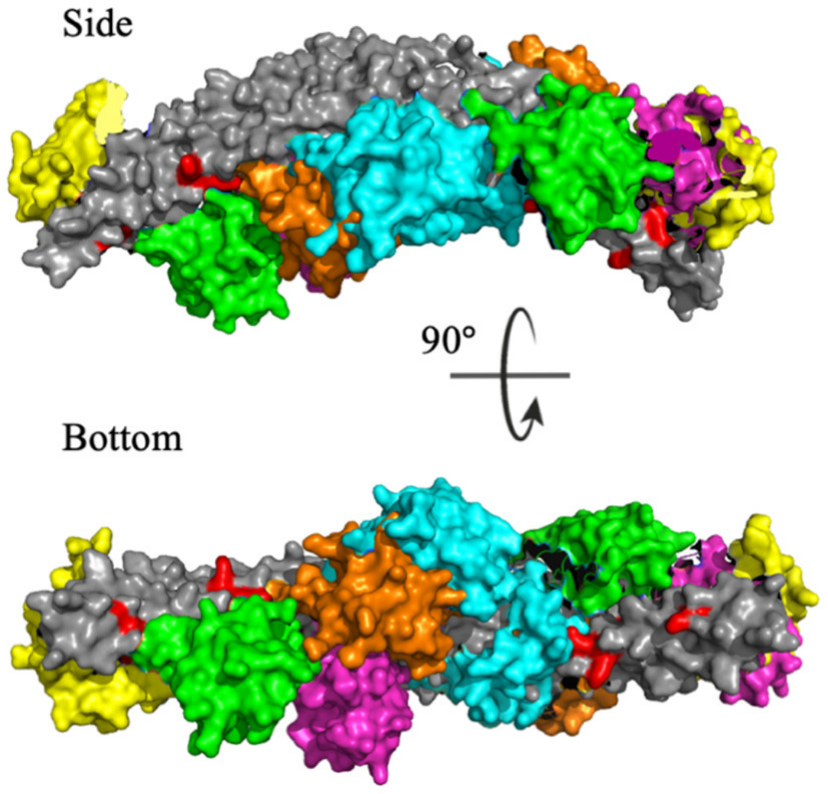
Cluster analysis reveals the most probable positions of the PDZ domains. The dimeric BAR domain is shown in gray and each cluster of the PDZ domains is shown in a unique color. Cluster 1 (yellow) represents 30.9% of the frames, Cluster 2 (pink) represents 20.8% of the frames, Cluster 3 (green) represents 18.9% of the frames, Cluster 4 (orange) represents 18.8% of the frames, and Cluster 5 (cyan) represent 10.5% of the frames. K251, K252, K257, K266, and K268 are colored red.

To identify the preferred regions on the dimeric BAR domain which interact with the PDZ domains, cluster analysis revealed the five most probable positions of the PDZ domains in space. Figure 6 portrays an overlay of these five clusters, where the dimeric BAR domain is shown in gray and each cluster is represented by a unique color of the PDZ domain. Furthermore, the five key positively charged residues on the concave surface of the BAR domain that readily interact with the surface of the lipid membrane are colored red. The most probable positions of the PDZ domain could physically block these key residues on the BAR domain from interacting with the membrane. While the most probable positions of the PDZ domains are on the concave surface of the BAR domain, the movement of the PDZ domains remains very dynamic. When the PDZ domains depart from the concave surface of BAR dimer, it may interact with the C-terminus of its binding partners and pull the BAR domain closer to the lipid membrane.

**Figure 7:**
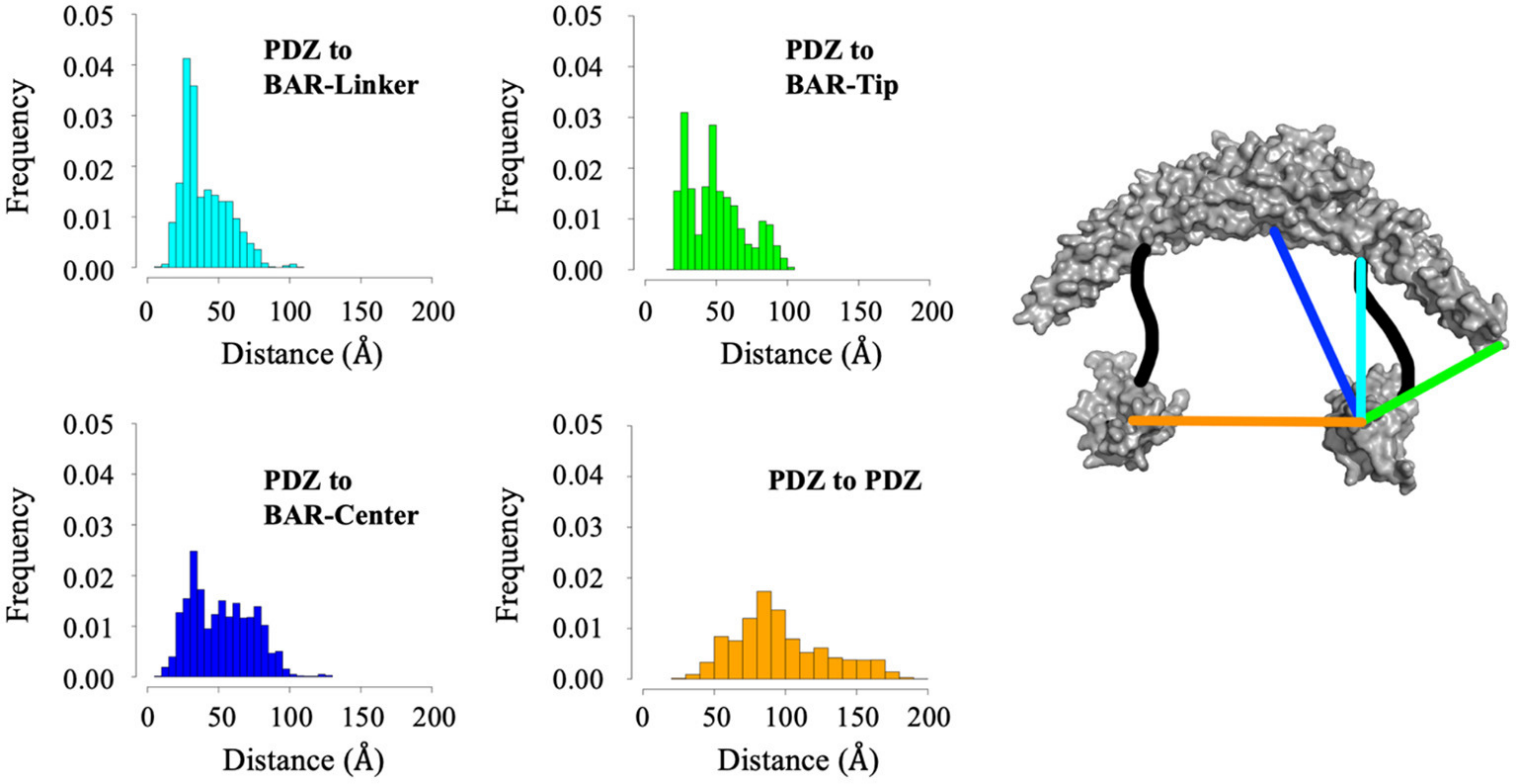
Distance analysis between the PDZ domain and the BAR domain. Distance between the PDZ domain and the BAR-Linker was defined by residues L60 and S130. Distance between the PDZ domain and the BAR-tip was defined by residues L60 and S262. Distance between the PDZ domain the BAR-Center was defined by residues L60 and T167. Distance between the two PDZ domains was defined by residues L60 and D390.

While cluster analysis reveals the most probable positions of the PDZ domain in respect to the BAR domain, RMSD and radius of gyration analysis reveal that the system has widely dynamic movements. In efforts to capture this range of motion and make a direct comparison to data reported by previous experiments (Karlsen et al., 2015), we performed centroid distance analysis as shown in Figure 7. Overall, our results agree with experimental data. The peak of the wide range of distances demonstrates the wide range of motion of the PDZ domain about the BAR domain. The major peaks of the distance distributions are for PDZ to BAR-Linker and PDZ to BAR-Tip reported by experiments was 20Å to 40Å, which agrees with our simulation data. In contrast, the distance between PDZ and BAR-Center does not precisely agree with experiments. Experimental data report the distance to be 60Å to 100Å while our simulations have shown a much broader distribution for this pair. For PDZ-PDZ distance, our simulations were able to capture the range corresponding to the range reported by experiments. It should be noted that while our simulations did produce a minor peak near 120Å that directly agrees with experiments, the overall distance distribution is shifted slightly to the left.

**Figure 8:**
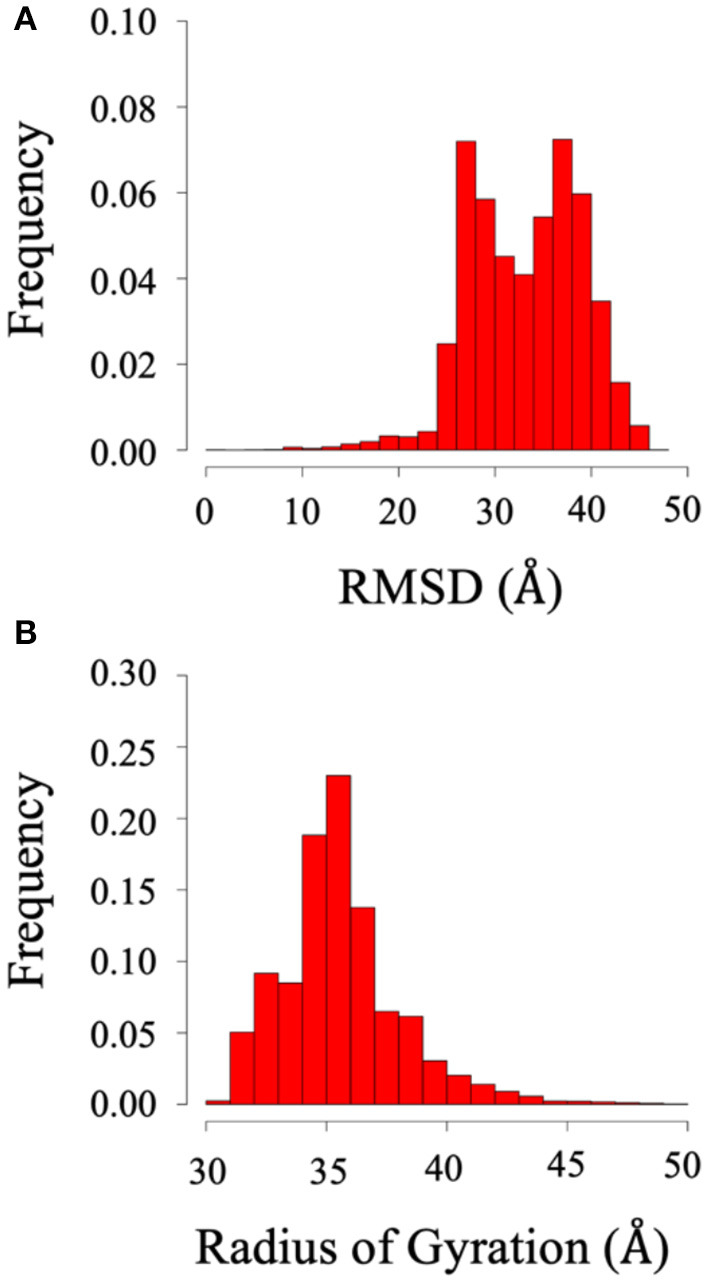
RMSD and radius of gyration of System 2 (BAR domain with C-termini). **(A)** RMSD. **(B)** Radius of gyration. The wide range of frequency signifies the system is very dynamic.

Root mean square deviation (RMSD) and radius of gyration (Rg) analysis was performed to quantify the flexibility of System 2 (the BAR domain with the C-termini), as shown in Figure 8. The RMSD plot (Figure 8A) shows the RMSD ranging from ~25 to 45 Å. This is a significant variation from the initial structure and demonstrates the wide range of motion of the C-termini. Furthermore, the radius of gyration analysis (Figure 8B) supports these results as the size of the protein fluctuates between 30 and 40 Å with relatively significant frequencies. This basic analysis reveals the wide range of motion of the C-terminal.

**Figure 9:**
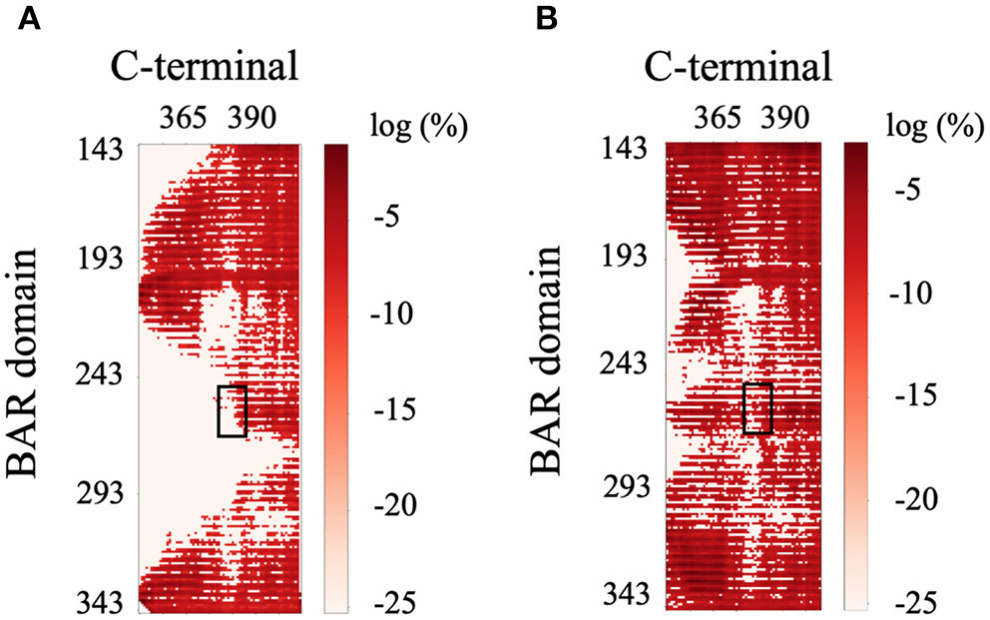
Contact map of BAR and C-terminus interactions. **(A)** C-terminus 1 and BAR 1 interactions. **(B)** C-terminus 1 and BAR 2 interactions. Black boxes indicated interactions between the key positively charged residues on the BAR domain (K251, K252, K257, K266, and K268) and the negatively charged residues of the C-terminus (D380-D389).

The previous literature (Jin et al., 2006) hypothesizes that the C-terminus negatively regulates the function of PICK1 by interacting with the key positively charged residues (K251, K252, K257, K266, and K268) on the concave surface of the BAR domain dimer that are critical to forming interactions with the lipid membrane. Interestingly, our results do not support these hypotheses. The C-termini are very dynamic and have a wide range of interactions with both with each other and the dimeric BAR domain as shown in Figure 9. The black boxes indicate contact between negatively charged stretch of residues that comprise the C-terminus (D380-D389) may form electrostatic interactions with the positively charged residues (K251, K252, K257, K266, and K268) on the BAR domains. These interactions formed contact in <1% of the frames with a separation of <8.0 Å. The two C-termini formed contact with each other as well. Most notably, the contact dissipates at the stretch of negatively charged residues (D380-D389).

While the C-terminus contains many charged residues, the driving forces guiding the interactions between the BAR and the C-terminus is unknown. Our work identifies the top ten pairs of residues forming interactions between the BAR domain and the C-terminus of PICK1. While the majority of identified pairs are driven by hydrophobic interactions, we also identified electrostatic interactions such as K209–D347. The high prevalence of hydrophobic interactions that are entropy driven may be due to the flexibility of the C-termini. All the contacts between the BAR domain and the C-terminus have a short average lifetime that is similar to the PDZ and linker interactions. Since the C-termini are flexible, the contacts between BAR and C-terminus form and break continuously.

**Figure 10:**
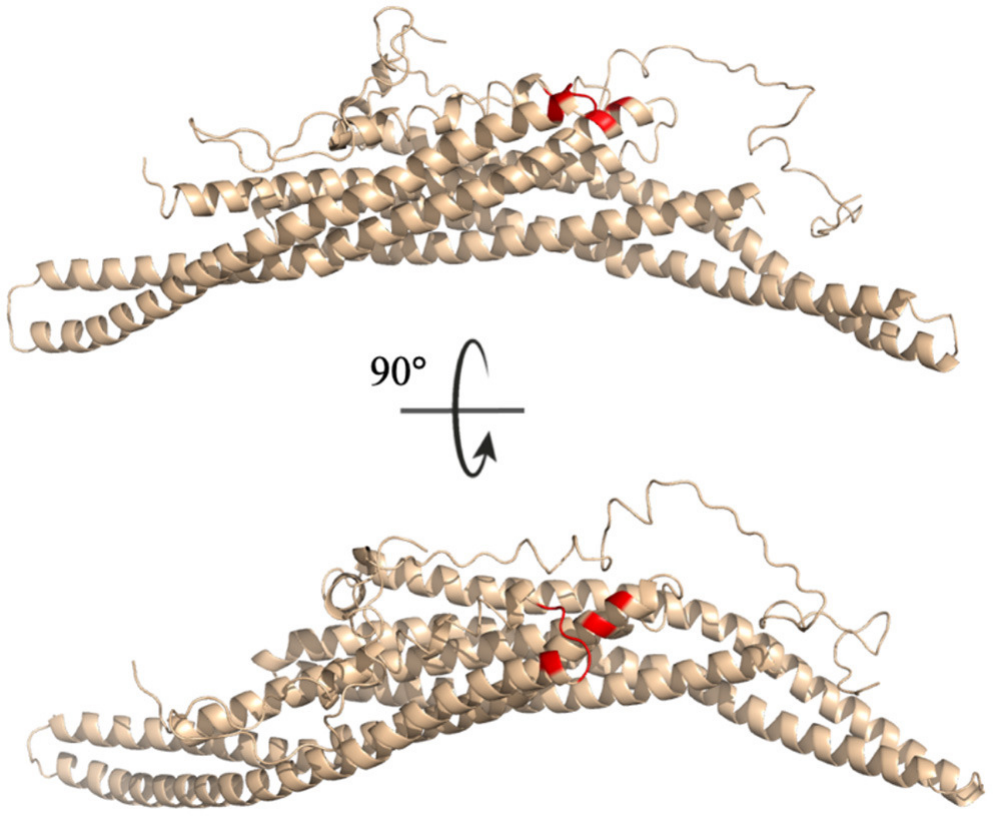
Key interaction pairs between the C-terminal and the BAR domain. All key residues listed in Table 3 have been colored red.

All residues identified in the top ten interaction pairs listed in Table 3 are highlighted in red in the BAR-C-termini structure shown in Figure 10. Potentially, these residues form the most probable interactions because of simple proximity. The BAR and C-termini most readily interacting at their connection site reinforces the notion of significantly flexibile C-termini.

**Table 3:** Interacting residue pairs between C-terminus and BAR domains.

**Residue 1**	**Residue type**	**Residue 2**	**Residue type**	**Probability (%)**	Lifetime^*^
341	MET	348	CYS	21.4	1.60 ± 1.10
342	SER	348	CYS	17.2	1.40 ± 0.80
209	LYS	347	ASP	17.0	1.31 ± 0.70
342	SER	347	ASP	16.6	1.26 ± 0.63
206	ALA	348	CYS	16.6	1.41 ± 0.89
341	MET	349	TYR	14.5	1.46 ± 0.85
342	SER	349	TYR	13.6	1.44 ± 0.84
210	PHE	348	CYS	13.5	1.38 ± 0.80
206	ALA	351	VAL	13.4	1.55 ± 1.03
340	THR	345	TYR	13.0	1.18 ± 0.53

* *unit is 100,000 UNRES simulation steps*.

**Figure 11:**
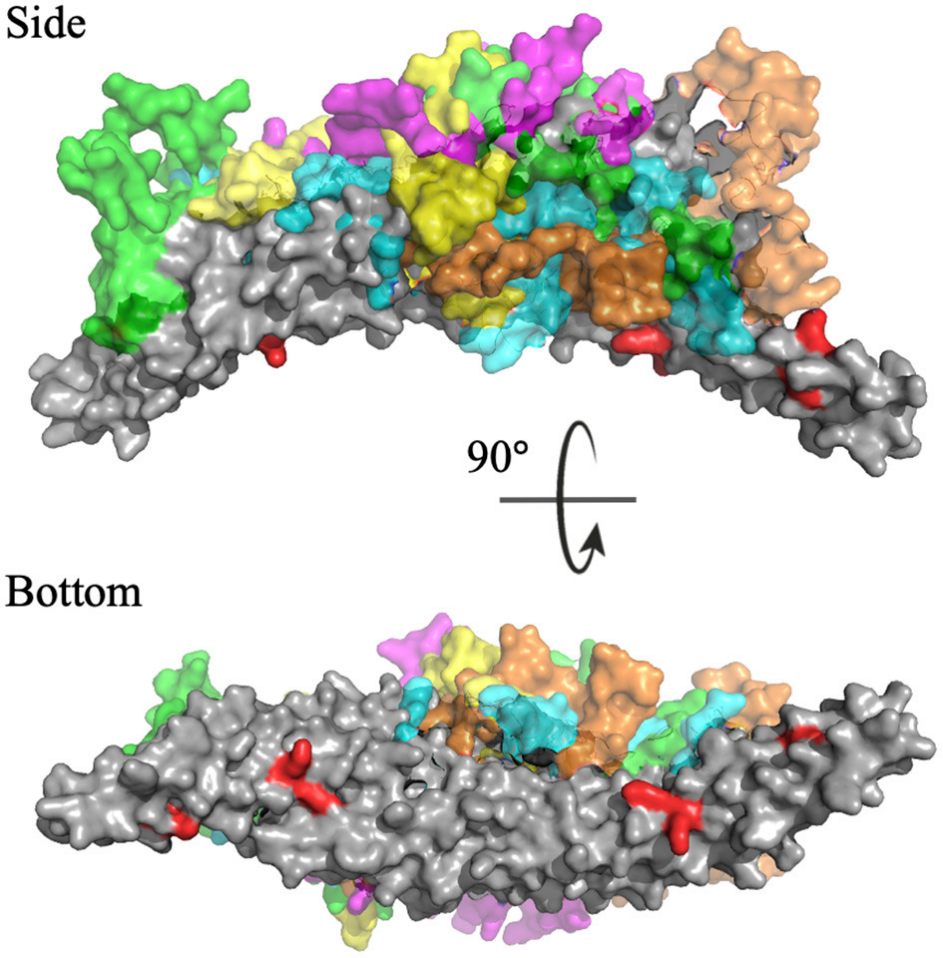
Cluster analysis reveals the most probable positions of the C-termini. The dimeric BAR domain is shown in gray and each cluster of the C-termini is shown in a unique color. Cluster 1 (purple) represents 66.6% of the frames, Cluster 2 (green) represents 38.9% of the frames, Cluster 3 (cyan) represents 22.3% of the frames, Cluster 4 (orange) represent 7.5% of the frames, and Cluster 5 (yellow) represents 4.6% of the frames. K251, K252, K257, K266, and K268 are colored red.

Cluster analysis revealed the five most probable positions of the C-termini in space. Figure 11 portrays an overlay of these five clusters, where the dimeric BAR domain is shown in gray and each cluster is represented by a unique color of the C-termini. Furthermore, the key positively charged residues on the concave surface of the BAR domain that readily interact with the surface of the lipid membrane are colored red. The most probable positions of the C-termini are centered on the convex surface of the dimeric BAR domain. The C-termini do not readily cover the key positively charged residues on the concave surface of the dimeric BAR domain as previously suspected. These results are in agreement with previous MD simulations of the PICK1 system (Salzer et al., 2017).

## Discussion

Our results demonstrate that the interdomain dynamics of PICK1 are driven by both electrostatic and hydrophobic interactions. Here, we identified key interaction pairs between the PDZ domain, linker, and dimeric BAR domain that are primarily hydrophobic interactions. While our results agree with previous experimental observations which suggest dynamic PDZ and BAR interaction patterns, the PDZ domain does have preferences on regions of interactions on the BAR domain. Interestingly, key residue interactions do not include the previously suspected positively charged residues (K251, K252, K257, K266, and K268) of the BAR domain but rather include neighboring residues. Surprisingly, the short helical fragment in the linker can form extensive interactions with the PDZ domain, potentially outcompeting the BAR domain. The biological function of the helical fragment may be more than just help to align to the BAR domain on the lipid membrane.

The interaction pairs demonstrate the significance of the βB-βC loop (Ala41, Gln42, and Tyr43) of the PDZ domain in initiating PDZ-BAR and PDZ-linker contact. Previous structural prediction via small-angle X-ray scattering (SAXS) analysis was unable to determine the orientation of the PDZ domain in PDZ-BAR interactions, but made the prediction that the βB-βC loop of the PDZ domain would orient toward the concave surface of the BAR domain (Madasu et al., 2015) Our simulations support this early hypothesis. Furthermore, previous literature reports the importance of the βB-βC loop in complex formation between the PDZ domain and activation ligand. Our previous work demonstrates the uniqueness of the PICK1 PDZ βB-βC loop (Stevens and He, 2020). A recent publication demonstrated a small-molecule inhibitor of the PICK1 PDZ domain with both strong affinity and specificity via targeting both the binding pocket and βB-βC loop of the PDZ domain (Christensen et al., 2020) Additionally, the βB-βC loop has been identified as an important player in PDZ-membrane interactions (Pan et al., 2007; Erlendsson and Madsen, 2015). Here, we show the relevance of the βB-βC loop in PDZ-BAR contact in the absence of an activating ligand. Key hydrophobic and electrostatic interactions between the PDZ domain and the BAR domain are initiated by residues that comprised the βB-βC loop. Furthermore, the interaction pairs reveal the significance of the βB-βC loop in initiating PDZ-BAR contact.

Previous experimental results (Jin et al., 2006) suggest that the C-terminus negatively regulates the function of PICK1 by physically covering the concave surface of the BAR domain dimer that interacts with the lipid membrane. The negatively charged stretch of residues that comprise the C-terminus (D380–D389) may form electrostatic interactions with the positively charged residues on the BAR domains that are critical in interactions with the negatively charged lipid bilayer. Interestingly, our results do not support these hypotheses. Our results demonstrate that the C-termini of PICK1 could directly interact with the positively charged residues (K251, K252, K257, K266, and K268) on the BAR domain, but actual interactions between these residues observed in our simulations are rare. We suspect that the C-termini may inhibit the higher-order aggregates of PICK1. PICK1 performs its biological function by forming clusters at the cell surface. Rather than covering key positively charged residues on the concave surface of the BAR domain, the C-termini may negatively inhibit the function of PICK1 by preventing scaffolding.

## Data Availability Statement

The raw data supporting the conclusions of this article will be made available by the authors, without undue reservation.

## Author Contributions

AS performed data analysis, carried out simulations, and wrote the manuscript. YH designed research strategy, performed data analysis, and wrote the manuscript.

## Conflict of Interest

The authors declare that the research was conducted in the absence of any commercial or financial relationships that could be construed as a potential conflict of interest.
